# Supplementary material for: Criterion-related validity and reliability of the Urdu version of the patient health questionnaire in a sample of community-based pregnant women in Pakistan
Source: PeerJ. 2018 Jul 17;6:e5185. doi: 10.7717/peerj.5185 (PMC6054083; doi:10.7717/peerj.5185)
Supplement: Supplemental Information 3 [file peerj-06-5185-s003.pdf]

SHARE/SHARECHILD SCREENING FORM

SECTION A: GREETING AND INTRODUCTION تعارف

Introduce yourself, HDRF, the project and the Screening Procedure

1.	Assessor Code ....انٹرویو کرنےوالے کا کوڈ	(S_SC_Assr_Code)
2.	Date of Interview DD/MM/YY...........انٹرویو کی تاریخ	(S_SC_DOI)

SECTION B: WOMAN'S INFORMATION عورت کی تفصیل

1	Woman's Name ____________________<br>خاتون کانام	(S_SC_Mo_Name)
2	Husband's Name ____________<br>خاوند کانام	(S_SC_Hb_Name)
3	UC Name/Code _____________________________<br>یو نین کونسل کا نام ، کوڈ	(S_SC_UC_Code)
4	Village Cluster Name/Code ___________________<br>گائوں کا نام	S_SC_Village_Code)
5	Household number ___________<br>گھرانہ نمبر	(S_SC_Hhld_no)

SECTION C: INFORMATION AND CONSENT معلومات اور رضامندی

Ensure that the Information sheet has been given or read out aloud to the woman

1	Have you read the information sheet or has it been read to you and you have understood it? آپ نے معلوماتی پرچہ پڑھا ہے یا آپ کو پڑھ کر سنایا گیا ہے۔اور آپ نے اس کو سمجھ لیا ہے؟	Yes<br>1 ہاں	No<br>0 نہیں	S_SC_infosheet
2	Do you agree to be part of the study?<br>کیا آپ اس تحقیق میں شمولیت اختیار کرنے کے لئے رضامند ہیں؟	Yes<br>1 ہاں	No<br>0 نہیں	S_SC_consent

SECTION D: ELIGIBILITY CRITERION اہلیت

1	What is your age (in years).<br>آپ کی عمر کتنی ہے؟ (سالوں میں)	_____________		S_SC_Mo_age
2	Are you married?<br>کیا آپ شادی شدہ ہیں؟	1: Married شادی شدہ<br>2: Separated علیحدگی ، خُلا<br>3: Divorced طلاق یافتہ<br>4. Widowed بیوہ<br>5. Unmarried غیر شادی شدہ		S_SC_mo_marital_status
3	Are you in your 3rd Trimester of pregnancy (5TH TO 8TH MONTH)?<br>کیاآپ حمل کی تیسری سہ ماہی میں ہیں؟(5 ماہ سے لے کر 8 ماہ تک)<br>(آپ کا کتنوا مہینہ چل رہا ہے؟)	Yes<br>1<br>ہاں	No<br>0<br>نہیں	S_SC_Mo_tri
	months	_____________		S_SC_Mo_tri_months
	days	_____________		S_SC_Mo_tri_days
4	Do you intend residing in the study area for at least ONE YEAR?<br>کیاآپ اس علاقے میں کم ازکم ایک سال تک رہیں گی؟	Yes<br>1 ہاں	No<br>0 نہیں	S_SC_Mo_stay
5	Can you speak & understand Urdu, Potohari, Punjabi and English?<br>کیا آپ اُردو، پوٹھوہاری، پنجابی اور انگریزی بول یا سمجھ سکتی ہیں؟	Yes<br>1 ہاں	No<br>0 نہیں	S_SC_Mo_lang
6	Note if the woman requires immediate inpatient care for any reason (medical or psychiatric). (Assessors Observation)	Yes<br>1 ہاں	No<br>0 نہیں	S_SC_mo_inp_care

SECTION E: PATIENT HEALTH QUESTIONNAIRE (PHQ-9) مریض کی صحت کے لئے سوالنامہ

Now I will be asking you questions about your health (physical & mental health). Now tell me “Over the last 2 weeks, how often have you been bothered by any of the following problems?
پچھلے دو ہفتوں کے دوران آپ کو مندرجہ ذیل مشکلات نے کتنی بار پریشان کیا.”

1	Feeling tired or having little energy.<br>تھکاوٹ اور جسمانی توانائی میں کمی محسوس ہوئی ۔	0---Not at all بالکل نہیں<br>1---Several days کئی دن<br>2--- More than half the days آدھے دنوں سے زیادہ<br>3--- Nearly every day تقریباً ہر روز	(S_SC_PHQ_lethargy)
2	Poor appetite or overeating.<br>بھوک کی کمی یا زیادتی۔	0---Not at all بالکل نہیں<br>1---Several days کئی دن<br>2--- More than half the days آدھے دنوں سے زیادہ<br>3--- Nearly every day تقریباً ہر روز	(S_SC_PHQ9_appetite)
3	Trouble falling or staying asleep, or sleeping too much.<br>صحیح نیند نہ آنا، نیند میں کمی یا زیادتی۔	0---Not at all بالکل نہیں<br>1---Several days کئی دن<br>2--- More than half the days آدھے دنوں سے زیادہ<br>3--- Nearly every day تقریباً ہر روز	(S_SC_PHQ9_sleep)
4	Moving speaking so slowly that other people could have noticed. Or the opposite being so fidgety or restless that you have been moving around a lot more than usual. بات چیت کام کاج میں سستی یا بے چینی جو دوسروں نے بھی محسوس کی ہو۔	0---Not at all بالکل نہیں<br>1---Several days کئی دن<br>2--- More than half the days آدھے دنوں سے زیادہ<br>3--- Nearly every day تقریباً ہر روز	(S_SC_PHQ9_psychomotor_retardation)
5	Trouble concentrating on things, such as reading the newspaper or watching television.<br>کام پر توجہ نہ دینا مثلاً چیزیں رکھ کے بھول جانا یا ہانڈی میں نمک ڈالنا بھول جانا۔	0---Not at all بالکل نہیں<br>1---Several days کئی دن<br>2--- More than half the days آدھے دنوں سے زیادہ<br>3--- Nearly every day تقریباً ہر روز	(S_SC_PHQ9_lossofconcentration)
6	Little interest or pleasure in doing things۔ کام میں دل نہ لگتا ہو۔<br>(پہلے جن کاموں میں دل لگتا تھا مگر اب نہیں لگتا)	0---Not at all بالکل نہیں<br>1---Several days کئی دن<br>2--- More than half the days آدھے دنوں سے زیادہ<br>3--- Nearly every day تقریباً ہر روز	(S_SC_PHQ9_lossofinterest)
7	Feeling down, depressed, or hopeless.<br>صحت گری گری رہتی ہو، خفگان ، دل خفا رہنا ، خفگی ، ذہنی دباو یا نا امید ہونا۔	0---Not at all بالکل نہیں<br>1---Several days کئی دن<br>2--- More than half the days آدھے دنوں سے زیادہ<br>3--- Nearly every day تقریباً ہر روز	(S_SC_PHQ9_lossofpleasure)
8	Feeling bad about yourself or that you are a failure or have let yourself or your family down۔<br>اپنا آپ برا لگنا یا ایسا محسوس ہونا کہ آپ اپنی اور اپنے خاندان کی امیدوں پر پورا نہیں اُتر پائیں ۔	0---Not at all بالکل نہیں<br>1---Several days کئی دن<br>2--- More than half the days آدھے دنوں سے زیادہ<br>3--- Nearly every day تقریباً ہر روز	(S_SC_PHQ9_guilt)
9	Thoughts that you would be better off dead, or of hurting yourself۔<br>خود کو نقصان پہنچانے کا سوچنا یا ایسا سوچنا کے مر جانا بہتر ہے۔	0---Not at all بالکل نہیں<br>1---Several days کئی دن<br>2--- More than half the days آدھے دنوں سے زیادہ<br>3--- Nearly every day تقریباً ہر روز	(S_SC_PHQ9_Suicidality)
10	If you had any of above problems, how difficult these problems made it for you to do your work, take care of things at home, or get along with other people? اگر آپکو ان مشکلات کا سامنا کرنا پڑا ہے تو ان کی وجہ سے گھر کے کام کاج، گھر کی چیزوں کی دیکھ بھال یا لوگو ں کے ساتھ ملنے جلنے میں کتنی مشکل محسوس ہوئی؟	بالکل مشکل نہیں ہوئی Not difficult at all: 1<br>تھوڑی مشکل ہوئی Somewhat difficult: 2<br>بہت مشکل ہوئی۔ Very difficult: 3<br>انتہائی مشکل ہوئی Extremely difficult: 4<br>لاگو نہیں ہوتا Not Applicable: 9	S_SC_PHQ9_difficulty

11	PHQ-9 Total Score	*Scores of 1 to 9 to be added*	(S_SC_PHQ9_Total)

SECTION F: SCID

CURRENT MAJOR DEPRESSIVE EPISODE

Now I would like to ask some questions about your mood

اب میں آپ کی جسمانی اور ذہنی صحت کے بارے میں کچھ سوال پو چھوں گی۔ پچھلے ایک ماہ کو ذہن میں رکھتے ہوئے مجھے جواب دیں۔

1. Depressed mood<br>طبعیت میں اداسی	9 --- inadequate information<br>1 --- No<br>2 --- Sub-threshold<br>3 --- Yes	SC_SCID_Depressedmood
2. Loss of interest<br>چیزوں میں عدم دلچسپی	9 --- inadequate information<br>1 --- No<br>2 --- Sub-threshold<br>3 --- Yes	SC_SCID_lossofinterest
3. Weight/ Appetite loss or gain<br>وزن اور بھوک کا بڑھنا یا گھٹنا<br>Probes: Weight loss or decreased appetite, Weight gain or increased appetite	9 --- inadequate information<br>1 --- No<br>2 --- Sub-threshold<br>3 --- Yes	SC_SCID_wtappetite_lossgain
4. Sleep disturbance<br>نیندکی خرابی<br>Probes: Insomnia, Hypersomnia	9 --- inadequate information<br>1 --- No<br>2 --- Sub-threshold<br>3 --- Yes	SC_SCID_sleepdisturbance
5. Psychomotor agitation or retardation<br>بے چینی یا سستی<br>Probes: Psychomotor agitation, Psychomotor retardation	9 --- inadequate information<br>1 --- No<br>2 --- Sub-threshold<br>3 --- Yes	SC_SCID_psychomotoragitation retardation
6. Fatigue or loss of energy<br>تھکا وٹ ، کمزوری	9 --- inadequate information<br>1 --- No<br>2 --- Sub-threshold<br>3 --- Yes	SC_SCID_fatigue
7. Feelings of worthlessness or inappropriate guilt<br>پچھتاوا یا احساس<br>ندامت<br>Probes: Worthlessness, Inappropriate guilt	9 --- inadequate information<br>1 --- No<br>2 --- Sub-threshold<br>3 --- Yes	SC_SCID_worthlessness
8. Diminished ability to concentrate or indecisiveness<br>فیصلہ اور تو جہ کرنے میں دشواری ہونا<br>Probes: Diminished ability to think, Indecisiveness	9 --- inadequate information<br>1 --- No<br>2 --- Sub-threshold<br>3 --- Yes	SC_SCID_indecisive
9. Recurrent thoughts of death or suicidal ideation<br>بار بار خود کشی کا خیا ل آنا<br>Probes: Thoughts of own death, Suicidal ideation, Specific plan, Suicide Attempt	9 --- inadequate information<br>1 --- No<br>2 --- Sub-threshold<br>3 --- Yes	SC_SCID_suicidalthoughts
10. Symptoms cause significant distress or impairment<br>یہ تمام علامات آپ کے لئے ذہنی دباو اور صحت کی خرابی کی و جہ بن رہے ہیں	9 --- inadequate information<br>1 --- No<br>2 --- Sub-threshold<br>3 --- Yes	SC_SCID_symptomsofdistress
11. Not due to direct effects of a substance or medical condition	9 --- inadequate information<br>1 --- No	SC_SCID_Effectsofsubstance_medicalconditon

کسی دوائی یا کسی جسما نی بیماری کا اثرتو نہیں	2 --- Sub-threshold<br>3 --- Yes	
12. Not better accounted for by bereavement<br>کسی کی موت کی وجہ سےتو نہیں	9 --- inadequate information<br>1 --- No<br>2 --- Sub-threshold<br>3 --- Yes	SC_SCID_bereavement
13. CURRENT MAJOR DEPRESSIVE EPISODE	9 --- inadequate information<br>1 --- No<br>2 --- Sub-threshold<br>3 --- Yes	SC_SCID_majordepressiveepiso de
14. Current MDE Date of onset:	___________________	SC_SCID_MDE_DOonset
DETAILED HISTORY OF RECURRENT MOOD EPISODES<br>Past major depressive episodes		
1. Have you ever felt like the previously asked questions within the past year?<br>اس سے پہلے جو کیفیات آپ نے بیان کی ہیں ،ایسی کیفیات آپ نے پچھلے سال میں کبھی محسوس کیں؟	0--- No<br>1--- Yes	SC_SCID_pastepisode
1a. If Yes, then how many times within the last year<br>اگر ہاں تو پچھلے سال میں کتنی بار	____________	SC_SCID_pastepisode_numbers
MAJOR DEPRESSIVE EPISODE A	0--- No<br>1--- Yes	SC_SCID_EpisodeA
Date of onset:	________________	SC_SCID_A_DOonset
Age:	________________	SC_SCID_A_age
Date of offset:	________________	SC_SCID_A_DOoffset
1. Depressed mood<br>طبعیت میں اداسی	9 --- inadequate information<br>1 --- No<br>2 --- Sub-threshold<br>3 --- Yes	SC_SCID_A_Depressedmood
2. Loss of interest<br>چیزوں میں عدم دلچسپی	9 --- inadequate information<br>1 --- No<br>2 --- Sub-threshold<br>3 --- Yes	SC_SCID_A_lossofinterest
3. Weight/ Appetite loss or gain<br>وزن اور بھوک کا بڑھنا یا گھٹنا<br>Probes: Weight loss or decreased appetite, Weight gain or increased appetite	9 --- inadequate information<br>1 --- No<br>2 --- Sub-threshold<br>3 --- Yes	SC_SCID_A_wtappetite_lossgai n
4. Sleep disturbance<br>نیندکی خرابی<br>Probes: Insomnia, Hypersomnia	9 --- inadequate information<br>1 --- No<br>2 --- Sub-threshold<br>3 --- Yes	SC_SCID_A_sleepdisturbance
5. Psychomotor agitation or retardation<br>بے چینی یا سستی<br>Probes: Psychomotor agitation, Psychomotor retardation	9 --- inadequate information<br>1 --- No<br>2 --- Sub-threshold<br>3 --- Yes	SC_SCID_A_psychomotoragitati onretardation
6. Fatigue or loss of energy<br>تھکا وٹ ، کمزوری	9 --- inadequate information<br>1 --- No<br>2 --- Sub-threshold	SC_SCID_A_fatigue

	3 --- Yes	
7. Feelings of worthlessness or inappropriate guilt<br>پچھتاوا یا احساس<br>ندامت<br>Probes: Worthlessness, Inappropriate guilt	9 --- inadequate information<br>1 --- No<br>2 --- Sub-threshold<br>3 --- Yes	SC_SCID_A_worthlessness
8. Diminished ability to concentrate or indecisiveness<br>فیصلہ اور تو جہ کرنے میں دشواری ہونا<br>Probes: Diminished ability to think, Indecisiveness	9 --- inadequate information<br>1 --- No<br>2 --- Sub-threshold<br>3 --- Yes	SC_SCID_A_indecisive
9. Recurrent thoughts of death or suicidal ideation<br>بار بار خود کشی کا خیا ل آنا<br>Probes: Thoughts of own death, Suicidal ideation, Specific plan, Suicide Attempt	9 --- inadequate information<br>1 --- No<br>2 --- Sub-threshold<br>3 --- Yes	SC_SCID_A_suicidalthoughts
10. Symptoms cause significant distress or impairment<br>یہ تمام علامات آپ کے لئے ذہنی دباو اور صحت کی خرابی کی و جہ بن رہے ہیں	9 --- inadequate information<br>1 --- No<br>2 --- Sub-threshold<br>3 --- Yes	SC_SCID_A_symptomsofdistres s
11. Not due to direct effects of a substance or medical condition<br>کسی دوائی یا کسی جسما نی بیماری کا اثرتو نہیں	9 --- inadequate information<br>1 --- No<br>2 --- Sub-threshold<br>3 --- Yes	SC_SCID_A_Effectsofsubstance _medicalconditon
12. Not better accounted for by bereavement<br>کسی کی موت کی وجہ سےتو نہیں	9 --- inadequate information<br>1 --- No<br>2 --- Sub-threshold<br>3 --- Yes	SC_SCID_A_bereavement
13. MAJOR DEPRESSIVE EPISODE	9 --- inadequate information<br>1 --- No<br>2 --- Sub-threshold<br>3 --- Yes	SC_SCID_A_majordepressiveepi sode
MAJOR DEPRESSIVE EPISODE B	0--- No<br>1--- Yes	SC_SCID_EpisodeB
Date of onset:	_______________	SC_SCID_B_DOonset
Age:	_______________	SC_SCID_B_age
Date of offset:	_______________	SC_SCID_B_DOoffset
1. Depressed mood<br>طبعیت میں اداسی	9 --- inadequate information<br>1 --- No<br>2 --- Sub-threshold<br>3 --- Yes	SC_SCID_B_Depressedmood
2. Loss of interest<br>چیزوں میں عدم دلچسپی	9 --- inadequate information<br>1 --- No<br>2 --- Sub-threshold<br>3 --- Yes	SC_SCID_B_lossofinterest
3. Weight/ Appetite loss or gain<br>وزن اور بھوک کا بڑھنا یا گھٹنا<br>Probes: Weight loss or decreased appetite, Weight gain or increased appetite	9 --- inadequate information<br>1 --- No<br>2 --- Sub-threshold<br>3 --- Yes	SC_SCID_B_wtappetite_lossgai n
4. Sleep disturbance	9 --- inadequate information	SC_SCID_B_sleepdisturbance

نیندکی خرابی<br>Probes: Insomnia, Hypersomnia	1 --- No<br>2 --- Sub-threshold<br>3 --- Yes	
5. Psychomotor agitation or retardation<br>بے چینی یا سستی<br>Probes: Psychomotor agitation, Psychomotor retardation	9 --- inadequate information<br>1 --- No<br>2 --- Sub-threshold<br>3 --- Yes	SC_SCID_B_psychomotoragitationretardation
6. Fatigue or loss of energy<br>تھکا وٹ ، کمزوری	9 --- inadequate information<br>1 --- No<br>2 --- Sub-threshold<br>3 --- Yes	SC_SCID_B_fatigue
7. Feelings of worthlessness or inappropriate guilt<br>پچھتاوا یا احساس<br>ندامت<br>Probes: Worthlessness, Inappropriate guilt	9 --- inadequate information<br>1 --- No<br>2 --- Sub-threshold<br>3 --- Yes	SC_SCID_B_worthlessness
8. Diminished ability to concentrate or indecisiveness<br>فیصلہ اور تو جہ کرنے میں دشواری ہونا<br>Probes: Diminished ability to think, Indecisiveness	9 --- inadequate information<br>1 --- No<br>2 --- Sub-threshold<br>3 --- Yes	SC_SCID_B_indecisive
9. Recurrent thoughts of death or suicidal ideation<br>بار بار خود کشی کا خیا ل آنا<br>Probes: Thoughts of own death, Suicidal ideation, Specific plan, Suicide Attempt	9 --- inadequate information<br>1 --- No<br>2 --- Sub-threshold<br>3 --- Yes	SC_SCID_B_suicidalthoughts
10. Symptoms cause significant distress or impairment<br>یہ تمام علامات آپ کے لئے ذہنی دباو اور صحت کی خرابی کی و جہ بن رہے ہیں	9 --- inadequate information<br>1 --- No<br>2 --- Sub-threshold<br>3 --- Yes	SC_SCID_B_symptomsofdistress
11. Not due to direct effects of a substance or medical condition<br>کسی دوائی یا کسی جسما نی بیماری کا اثرتو نہیں	9 --- inadequate information<br>1 --- No<br>2 --- Sub-threshold<br>3 --- Yes	SC_SCID_B_Effectsofsubstance_medicalconditon
12. Not better accounted for by bereavement<br>کسی کی موت کی وجہ سےتو نہیں	9 --- inadequate information<br>1 --- No<br>2 --- Sub-threshold<br>3 --- Yes	SC_SCID_B_bereavement
13. MAJOR DEPRESSIVE EPISODE	9 --- inadequate information<br>1 --- No<br>2 --- Sub-threshold<br>3 --- Yes	SC_SCID_B_majordepressiveepisode
MAJOR DEPRESSIVE EPISODE C	0--- No<br>1--- Yes	SC_SCID_EpisodeC
Date of onset:	_______________	SC_SCID_C_DOonset
Age:	_______________	SC_SCID_C_age
Date of offset:	_______________	SC_SCID_C_DOoffset
1. Depressed mood<br>طبعیت میں اداسی	9 --- inadequate information<br>1 --- No<br>2 --- Sub-threshold	SC_SCID_C_Depressedmood

	3 --- Yes	
2. Loss of interest<br>چیزوں میں عدم دلچسپی	9 --- inadequate information<br>1 --- No<br>2 --- Sub-threshold<br>3 --- Yes	SC_SCID_C_lossofinterest
3. Weight/ Appetite loss or gain<br>وزن اور بھوک کا بڑھنا یا گھٹنا<br>Probes: Weight loss or decreased appetite, Weight gain or increased appetite	9 --- inadequate information<br>1 --- No<br>2 --- Sub-threshold<br>3 --- Yes	SC_SCID_C_wtappetite_lossgain
4. Sleep disturbance<br>نیندکی خرابی<br>Probes: Insomnia, Hypersomnia	9 --- inadequate information<br>1 --- No<br>2 --- Sub-threshold<br>3 --- Yes	SC_SCID_C_sleepdisturbance
5. Psychomotor agitation or retardation<br>بے چینی یا سستی<br>Probes: Psychomotor agitation, Psychomotor retardation	9 --- inadequate information<br>1 --- No<br>2 --- Sub-threshold<br>3 --- Yes	SC_SCID_C_psychomotoragitationretardation
6. Fatigue or loss of energy<br>تھکا وٹ ، کمزوری	9 --- inadequate information<br>1 --- No<br>2 --- Sub-threshold<br>3 --- Yes	SC_SCID_C_fatigue
7. Feelings of worthlessness or inappropriate guilt<br>پچھتاوا یا احساس<br>ندامت<br>Probes: Worthlessness, Inappropriate guilt	9 --- inadequate information<br>1 --- No<br>2 --- Sub-threshold<br>3 --- Yes	SC_SCID_C_worthlessness
8. Diminished ability to concentrate or indecisiveness<br>فیصلہ اور تو جہ کرنے میں دشواری ہونا<br>Probes: Diminished ability to think, Indecisiveness	9 --- inadequate information<br>1 --- No<br>2 --- Sub-threshold<br>3 --- Yes	SC_SCID_C_indecisive
9. Recurrent thoughts of death or suicidal ideation<br>بار بار خود کشی کا خیا ل آنا<br>Probes: Thoughts of own death, Suicidal ideation, Specific plan, Suicide Attempt	9 --- inadequate information<br>1 --- No<br>2 --- Sub-threshold<br>3 --- Yes	SC_SCID_C_suicidalthoughts
10. Symptoms cause significant distress or impairment<br>یہ تمام علامات آپ کے لئے ذہنی دباو اور صحت کی خرابی کی و جہ بن رہے ہیں	9 --- inadequate information<br>1 --- No<br>2 --- Sub-threshold<br>3 --- Yes	SC_SCID_C_symptomsofdistress
11. Not due to direct effects of a substance or medical condition<br>کسی دوائی یا کسی جسما نی بیماری کا اثرتو نہیں	9 --- inadequate information<br>1 --- No<br>2 --- Sub-threshold<br>3 --- Yes	SC_SCID_C_Effectsofsubstance_medicalconditon
12. Not better accounted for by bereavement<br>کسی کی موت کی وجہ سےتو نہیں	9 --- inadequate information<br>1 --- No<br>2 --- Sub-threshold<br>3 --- Yes	SC_SCID_C_bereavement
13. MAJOR DEPRESSIVE EPISODE	9 --- inadequate information<br>1 --- No	SC_SCID_C_majordepressiveepisode

	2 --- Sub-threshold<br>3 --- Yes	

SECTION G: INFORMING ABOUT THE DIAGNOSIS & REFERRAL تشخیص اور ریفریل کے بارے میں معلومات

Inform the woman about her diagnosis and how to seek care (EUC). Also inform her that she will be provided with a referral letter for IoP, Rawalpindi.

1	Has the woman been informed of her diagnosis ? آپ نے جو تشخیص کی ہے اس کے بارے میں عورت کومعلوم ہے؟	Yes: 1 ہاں<br>No: 0 نہیں	S_SC_diag
2	Has the woman been provided information on how to seek care (EUC)? کیا آپ نے عورت کو معلومات دی ہیں که وہ کہاں سے مدد حاصل کرے؟	Yes: 1 ہاں<br>No: 0 نہیں	S_SC_EUC_info
3	Has the referral letter been given to the woman?<br>کیا عورت کو ریفریل پرچه دیا گیا ہے؟	Yes: 1 ہاں<br>N/A: 9 لاگو نہیں ہوتا<br>No: 0 نہیں	S_SC_Referred

SECTION H: CONTACT DETAILS رابطے کی تفصیلات

Ask for the contact details of the participant and explain why these are being sought

1	Landline # لینڈ لائن نمبر	+ 92 51……..	(S_SC_Cntct_no_landline
2	Cell # موبائل نمبر	+923…..	S_SC_Cntct_no_cell)

SECTION I: CLOSE AND GPS جی پی ایس

Say you will see the woman again at after 3 months post-delivery When you leave the house/ interview if you have filled out details, take a GPS reading. Ensure you have a clear site of the sky and take no more than 10 paces from the house.

GPS coordinates				.	N	S_SC_HH_LATITUDE
				.	E	S_SC_HH_LONGITUDE
